# Total and Compartmental Chest Wall Volumes, Lung Function, and Respiratory Muscle Strength in Individuals with Abdominal Obesity: Effects of Body Positions

**DOI:** 10.1155/2019/9539846

**Published:** 2019-12-16

**Authors:** Rattanaporn Sonpeayung, Anong Tantisuwat, Prawit Janwantanakul, Premtip Thaveeratitham

**Affiliations:** Department of Physical Therapy, Faculty of Allied Health Sciences, Chulalongkorn University, Bangkok 10330, Thailand

## Abstract

**Background:**

Abdominal obesity is a chronic condition that can contribute to impairments in lung function, leading to increased risks for respiratory-related diseases. Body position is an important technique that effectively restores and increases lung function and chest wall volumes. The objective of the current study was to examine the effects of the body positions on total and compartmental chest wall volumes, lung function, and respiratory muscle strength in individuals with and without abdominal obesity.

**Methods:**

Twenty obesity and twenty healthy males performed in four body position including sitting without and with back support, Fowler's, and supine positions. Each position was performed for five minutes. Chest wall volumes, lung function, and respiratory muscle strength were assessed in each position.

**Results:**

Sitting without and with back support resulted in higher total and rib cage compartmental chest wall volumes, lung function, and inspiratory muscle strength than Fowler's and supine positions in both groups (*p* < 0.001). Abdominal obesity subjects had significantly less total and compartmental chest wall volumes and lung function across four body positions than healthy subjects (*p* < 0.001). Respiratory muscle strength in the obesity group was less than that in the healthy control group (*p* > 0.05).

**Conclusions:**

This study provides new information regarding the effect of obesity and body position on chest wall volumes, lung function, and respiratory muscle strength. Among obesity individuals who are bedridden, sitting increases lung function, total and rib cage compartmental chest wall volumes, and inspiratory muscle strength—and would therefore likely to decrease the risk of respiratory-related disease—relative to Fowler's and supine positions.

## 1. Introduction

Impairments of lung function, chest wall motion, and dysfunctional breathing are common in patients with cardiopulmonary problems [1–3]. Abdominal obesity is a chronic disorder associated with a high risk for developing noncommunicable diseases (NCDs) such as diabetes mellitus, hypertension, heart disease, metabolic syndrome, respiratory diseases, and mortality rate [4, 5]. Abdominal obesity-related illnesses—in particular respiratory diseases—cost the healthcare system in Thailand more than 35 million US dollars annually [6]. Obesity is also known to contribute to impairments in lung function, breathing patterns, and the development of respiratory complication and diseases [1–3]. Excessive fat accumulation in the thoracic-abdominal region restricts the chest wall expansion and diaphragmatic muscle contraction, lengthens abdominal muscles, reduces the upper airway calibre, modifies airway configuration, and increases in intra-abdominal pressure [1–3, 7–10]. Rapid and shallow breathing in abdominal obesity individuals has also been shown to increase risk for hypoxia, airflow limitations, breathing workload, and the development of respiratory disease, medical complications, and longer hospital stays after surgery [11, 12].

There were many factors that can influence lung function, such as age, sex, physical activity, and body positions [1–3, 7–10]. Of these, body position changes can be used as an intervention to improve lung function [13–16], given the direct effect of body position on chest wall motion, respiratory muscles performance, and breathing patterns [13–17], including in individuals with obesity [18–21]. There are a variety of body positions that are usually used in this context, including sitting, Fowler's, side lying, supine, and prone positions for preserving and restoring lung performance and reducing the risk of respiratory complications [14, 15, 22].

The studies have shown that changes from a sitting to a supine position has reduced lung function, including total lung capacity (TLC), force vital capacity (FVC), force expiratory volume in first second (FEV1), FEV1/FVC, peak expiratory flow (PEF), expiratory reserve volume (ERV), and functional residual capacity (FRC) in obesity individuals [18–21, 23]. However, these studies focused only on sitting and supine positions. On the other hand, Benedik's study investigated the other positions (Fowler's positions). They found that there was no difference in FRC between Fowler's and supine positions in individuals with mild to moderate obesity [23]. The body positions have influenced not only on lung function and respiratory muscle strength but also the different compartments of chest wall volume and diameter changes in obesity individuals [24, 25]. The chest wall compartments comprise rib cage (RC) and abdomen (AB) compartments. The RC was divided into pulmonary and abdominal rib cage compartments [26]. With respect to the chest wall volume, there was only Barcelar's study which investigated the effect of sitting position on chest wall volume and its compartments in individuals with obesity [24]. The results showed that obesity significantly reduced the pulmonary rib cage chest wall volume compared with the control group in sitting position [24]. Regarding the chest wall diameter changes, Sonpeayung and colleagues reported that obesity individuals significantly decreased the chest wall diameter changes across body positions relative to the control group (*p* < 0.01) [25].

Although the research performed to date in this area indicates an effect of obesity and body positions on a number of measures of lung function, no research has systematically examined the effects of both abdominal obesity and multiple body positions on measures of lung function and chest wall volumes in the same group of individuals. For example, the evidence [18–21, 23] on lung function had studied only sitting versus supine positions without specific type of obesity, and only one study examined effects of Fowler's position on lung function (functional residual capacity outcome) [23]. There is only one study which examined the effect of sitting positions on chest wall volumes [24]. To address these knowledge gaps, here we examined the effects of four body positions (sitting without and with back support, Fowler's, and a supine position) on multiple lung function domains (total and compartmental chest wall volumes, lung function, and respiratory muscle strength) in individuals with abdominal obesity. Based on the research performed to date, we hypothesized that males with abdominal obesity would evidence lower scores across all measures of lung function than nonobesity males. We did not have any a priori hypotheses regarding possible differences between sitting versus Fowler's position on lung function measures. In addition, we also anticipated that both sitting positions and Fowler's position would result in lung function improvement relative to supine position.

## 2. Materials and Methods

This study was approved by the Ethics Review Committee for Research Involving Human Research Subjects, Health Science Group, Chulalongkorn University (ERCCU) (Approval No. 052/2017). All subjects were informed about the research objectives and methods and written consent was obtained prior to data collection.

### 2.1. Study Population

The subjects were recruited from the general population through social media, study information brochures, and an announcement board in the university. A pilot study was conducted with ten men (5 obesity, 5 healthy control) to compute effect size estimates for the effects of obesity on the lung function measures (Cohen's *d* range, 0.43 to 0.47). With the most conservative of these (i.e., *d* = 0.47), a total of 40 subjects would be needed to provide sufficient power (80%) to detect difference between group differences.

The inclusion criteria included being a Thai male, being aged between 20–40 years old, not having any underlying health conditions, and being willing to undergo the study. Overall physical activity of subject was in the sedentary range, based on an average Baecke habitual physical activity score of less than 6 [27]. The subjects were classified into obesity and healthy control groups based on their body mass index (BMI), weight-hip ratio (WHR), and skinfold thickness. The healthy control group had BMI: 18.5–22.9 kg/m^2^, WHR: <0.9, and skinfold thickness: <90 mm in the normal ranges [24]. These scores in the obesity group were 25–34.99 kg/m^2^ (BMI) (8, 24), >0.9 (WHR) [24], and >90 mm (skinfold thickness) which represent abdominal obesity [28].

### 2.2. Procedure

**Figure 1:**
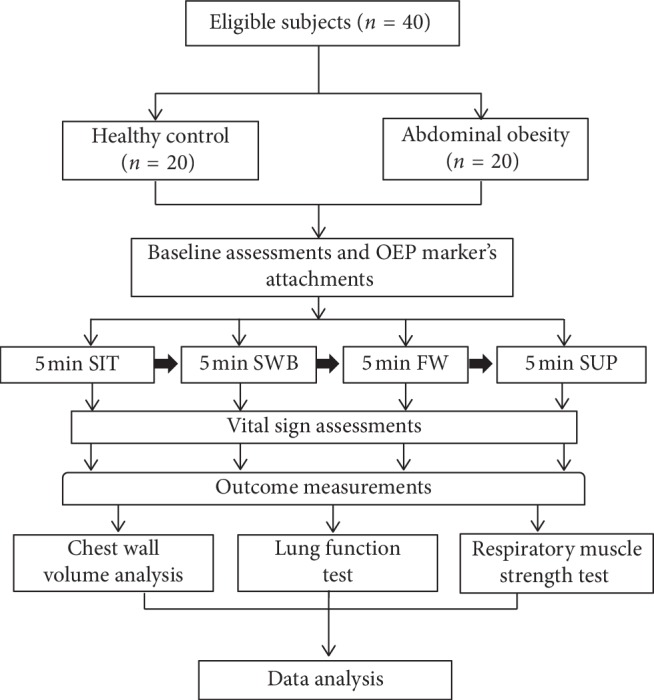
Flow diagram of the research procedure. SIT, sitting without back support; SWB, sitting with back support; FW, Fowler's position; SUP, supine position.

Research procedures are illustrated in Figure 1.

### 2.3. Baseline Assessments

Subject's baseline assessments were composed of anthropometric measurements, body compositions, and truncal skinfold thickness.

Anthropometric measurements including height, weight, and waist and hip circumferences were taken according to WHO expert consultation, [29]. Weight and height were measured using a stadiometer with shoes and heavy clothes removed [29]. Waist and hip circumference were determined by tape measure [29]. Both BMI (kg/m^2^) and WHR were calculated. Percentage of body fat, subcutaneous fat, and visceral fat were assessed via bioelectrical impedance (Karada scan: OMRON, Model HBF-375).

Truncal skinfold thickness was assessed at five sites (pectoral, midaxillary, subscapular, suprailiac, and abdomen sites) while subjects were standing using a digital outside skinfold caliper (Moore and Wright, UK) [28]. Three skinfold thickness measurements were performed at each site and averaged, and these averages were then summed to compute an overall skinfold thickness score [28].

### 2.4. Reflexive Marker Placements

In order to assess chest wall volume parameters, the subjects were attached reflexive markers around their chest wall for the three-dimensional (3D) chest wall analysis. The protocol for positioning the reflective markers has been previously described [30, 31]. Eighty-nine markers were used to assess chest wall volumes during the sitting without back support position [30, 31], and fifty-two markers were used to assess chest wall volumes during the sitting with back support, Fowler's, and supine positions [30, 31].

### 2.5. Body Positions

After all of the reflexive markers were placed, subjects performed four body positions including sitting without back support (SIT), sitting with back support (SWB), Fowler's, and supine positions for 5 minutes. Blood pressure, heart rate, respiratory rate, and oxygen saturation were assessed in each position to ensure that they remained stable. The sequence of the body position was SIT, SWB, Fowler's, and supine positions, respectively, in order to minimize small airway collapse from body weight during the lying position. Details regarding the four body positions are described as follows.

 
*SIT* involved sitting upright on the chair without a backrest, with 90 degrees of trunk inclination. Hip and knee positions were flexed at 90 degrees, and hip abduction was less than 10 degrees.
 
*SWB* involved sitting upright on the chair with padded foam pillow at the subject's shoulder level, with 90 degrees of trunk inclination. Hip and knee joints were that same as the SIT position.
 
*Fowler's position* involved semisitting position on a bed, with 45 degrees of trunk inclination. Hips and knees were slightly flexed about 15 degrees, with the support of a pillow.
 
*Supine position* involved lying horizontally on the bed. Head and neck positions were in a neutral position and supported by a pillow. Hips and knees were the same as the Fowler's position.

### 2.6. Outcome Measures

#### 2.6.1. Chest Wall Volumes

Optoelectronic plethysmography (OEP-BTS®, Milan, Italy) is a reliable and valid tool to measure 3D chest wall kinematics. The system is comprised of eight infrared light video cameras with sampling rate at 60 Hz. The measurement properties, operation principles, and calibration procedure have been previously described [30, 31]. Volume was calculated based on the surface triangulation of the 3D coordinates of the markers, using Gauss' theorem [31, 32].

OEP data were recorded during 5 minutes of quiet breathing in each body position. The subjects breathed spontaneously and avoided speaking or moving during the recording. The OEP system captures and tracks the 3D movement of the chest wall using the SMART TRACKER program. The MATLAB® software program version 2018a (The MathWorks Inc., Natick, MA, USA) was used to compute chest wall volume outcomes, including total chest wall volume (Vtot) as well as compartmental chest wall volumes including two rib cage volumes (Vrc) (pulmonary rib cage volume (Vrcp) and abdominal rib cage volume (Vrca)) and abdomen volume (Vab) [30, 31]. Vrc and Vab were recorded both as absolute values and as percentage contributions to total chest wall volume.

#### 2.6.2. Lung Function

Lung function was assessed in each body position using a computer spirometer (KOKO® spirometer, nSpire Health, Inc, USA). The spirometry system was calibrated prior to each assessment, based on the manufacturer's specifications. Room temperature was set optimally at 24–25 degrees Celsius. The researcher first demonstrated the protocol, and subjects then practiced two or three trials to ensure that they practiced correctly before true testing, based on the American Thoracic Society/European Respiratory Society recommendations [33]. Subjects were asked to take deep maximal inhalation and then to exhale forcefully as much as they could and for as long as possible using a mouth piece [33]. Lung function outcomes included FVC, FEV1, FEV1/FVC, PEF, and ERV. FVC, FEV1, FEV1/FVC, and PEF were measured by using FVC manoeuvre in the spirometry test. ERV was calculated by using SVC manoeuvre in the spirometry test. The highest value from three tests that had a difference of less than 10% among them was used as the lung function score [33].

#### 2.6.3. Respiratory Muscle Strength

A respiratory pressure meter (Micro RPM®, CareFusion, United Kingdom) was used to measure respiratory muscle strength. The maximum inspiratory pressure (MIP) was measured from residual volume to total lung capacity and maximum expiratory pressure (MEP) started from total lung capacity to residual volume based on the ATS/ERS guidelines [34]. Subjects were asked to complete trials until three occurred that has a less than 10% variation between them. The highest scores from the three trials were used for the measures of respiratory muscle strength.

### 2.7. Data Analysis

Data were analysed with statistics software (SPSS 22.0, Chicago, Illinois). Mean and standard deviation of all relevant variables were computed. Kolmogorov–Smirnov tests were used to verify normal distribution of the data. Independent *t*-test was used to compare the baseline characteristics between healthy control and abdominal obesity groups. Two-way repeated ANOVA with Bonferroni post hoc analyses evaluated the differences between two groups (healthy control and abdominal obesity groups). The level of significance was *p* < 0.05.

## 3. Results

### 3.1. Baseline Characteristics

The subjects' baseline characteristics are shown in Table 1. There were statistically significant differences in weight, BMI, waist and hip circumference, WHR, percentage of body fat and visceral fat, truncal skinfold thickness, and respiratory rate between healthy control and obesity groups (*p* < 0.0001).

### 3.2. Effects of Body Positions on Chest Wall Volume in Abdominal Obesity

**Figure 2:**
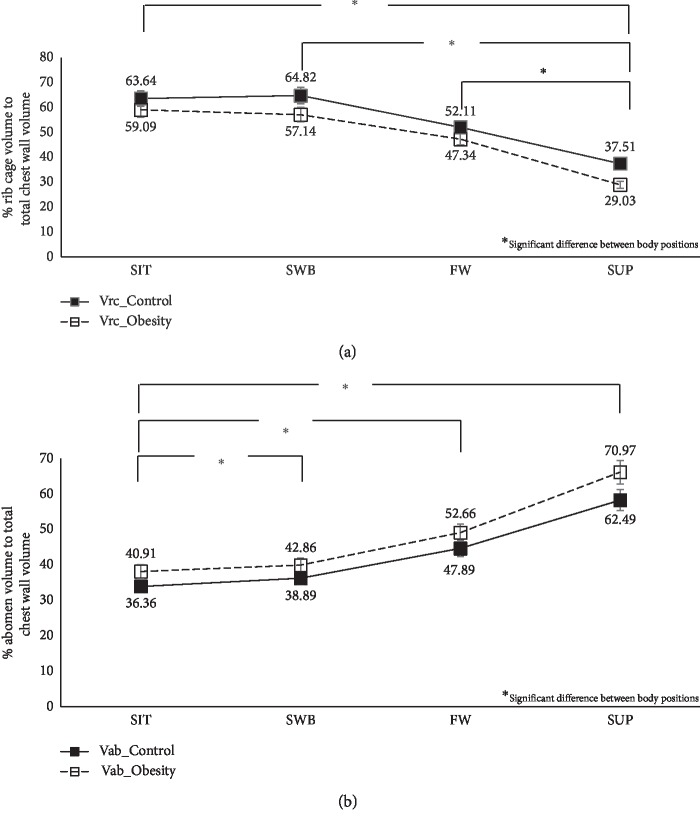
Effects of the body positions on % compartmental volume to total chest wall volume: (a) %Vrc; (b) %Vab.

**Figure 3:**
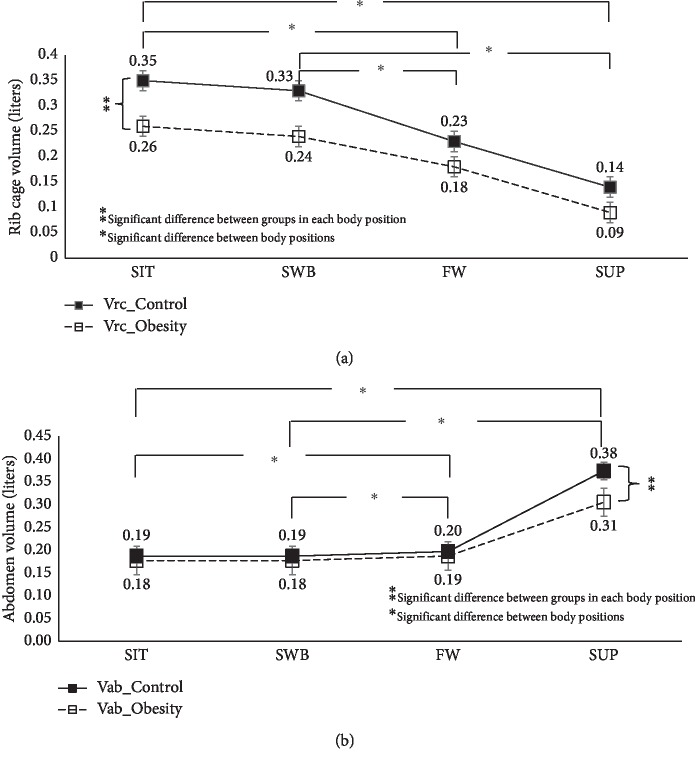
Effects of the body positions on chest wall volume: (a) Vrc; (b) Vab.

Figures 2 and 3 show the effects of body position on total and compartmental chest wall volumes in abdominal obesity. Comparing the four body positions, the supine showed the lowest Vtot. In addition, both SIT and SWB had significantly higher Vrc and %Vrc compared with both lying positions (Fowler's and supine positions) (*p* < 0.001).

### 3.3. Comparison between Abdominal Obesity and Control Groups across Body Positions on Chest Wall Volume

The abdominal obesity group had significantly less total and compartmental chest wall volumes across the different body positions than the healthy control group (*p* < 0.001) (Figures 2 and 3).

### 3.4. Effects of Body Positions on Lung Function in Abdominal Obesity

Table 2 shows the effects of abdominal obesity and body positions on lung function. In the abdominal obesity group, supine showed the lowest lung function parameters, followed by Fowler's position and SIT and SWB positions (*p* < 0.001).

### 3.5. Comparison between Abdominal Obesity and Control Groups across Body Positions on Lung Function

The abdominal obesity group had significantly decreased lung functions (FVC, FEV1, FEV1/FVC, PEF, and ERV) across all of the body positions than the healthy control group (*p* < 0.001) (Table 2).

### 3.6. Effects of Body Positions on Respiratory Muscle Strength in Abdominal Obesity

**Figure 4:**
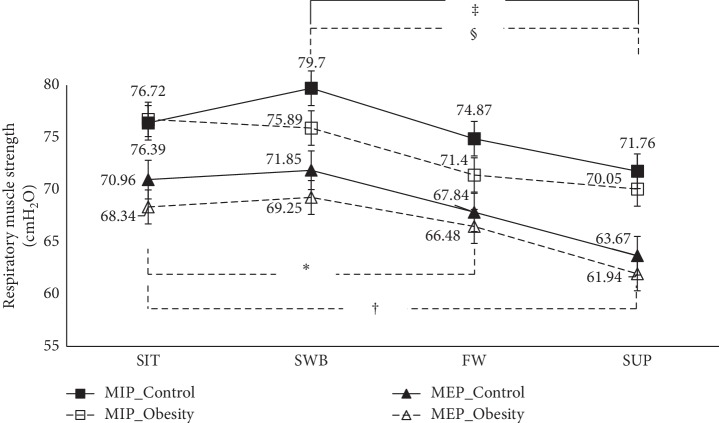
Effects of the body positions on MIP and MEP. ^*∗*^Significant difference of MIP between SIT and FW in the abdominal obesity group; ^†^significant difference of MIP between SIT and SUP in the abdominal obesity group; ^‡^significant difference of MIP between SWB and SUP in the abdominal obesity group; ^§^significant difference of MIP between SWB and SUP in the control group.

The results regarding the effects of body positions on respiratory muscle strength in the abdominal obesity group are presented in Figure 4. In the abdominal obesity group, both lying positions (FW and SUP) significantly had reduced MIP compared with the SIT position (*p* < 0.001). SWB had significantly increased MIP relative to supine position (*p* < 0.001).

### 3.7. Comparison between Abdominal Obesity and Control Groups across Body Positions on Respiratory Muscle Strength

No significant differences in respiratory muscle strength were observed across the body positions between the abdominal obesity and healthy control groups (Figure 4).

## 4. Discussion

The objective of the study was to test hypotheses regarding the impact of obesity and different body positions on chest wall volumes, lung function, and respiratory muscle strength. In comparisons between body positions, the current results indicated that both sitting positions in the abdominal obesity group showed the largest of the total and rib cage compartmental chest wall volume, lung function, and inspiratory muscle strength, followed by Fowler's and the supine position. Considering compartmental chest wall volumes, SIT position showed higher pulmonary ribcage volume and SWB position had greater abdominal ribcage volume than other body positions in the abdominal obesity group. In comparison with the control, the findings indicated 9% to 23% reductions in chest wall volumes and 12% to 24% reductions in lung function across the body positions in the abdominal obesity subjects, relative to the healthy control subjects. With respect to respiratory muscle strength, the abdominal obesity group had lower respiratory muscle strength than the healthy control group. All of changes in abdominal obesity group resulted from individuals having abdominal obesity without any health problems.

According to guidelines for pulmonary function tests, Thoracic Society of Thailand under Royal Patronage (2002), the current study has shown that individuals with abdominal obesity have restrictive lung defects by reducing FVC (87% of predicted) and FEV1/FVC (82% of predicted) relative to percentage of Thai normal value [35]. Furthermore, previous research has shown that obesity individuals tend to have a small airway collapse as reflected by a reduced ERV, relative to nonobesity individuals [36]. Interestingly, a reduction of lung function by even as little as one quarter (12% to 24% reduction was found in this study) may increase the chance to develop respiratory problems and complications in otherwise asymptomatic individuals with obesity [37]. Beeckman and colleagues reported that rapid declines in FEV1 of more than 15% is associated with a two-fold increase in the risk of dying of cardiovascular and nonmalignant respiratory diseases and a 3.2-fold greater risk of dying of chronic obstructive pulmonary disease [37]. Jakeways and colleagues found that reduced levels of FEV1 and FVC greater than 20% is associated with a two-fold risk of the development of chronic respiratory symptoms (e.g., cough, wheeze, and difficulty of breathing) in general population [38].

### 4.1. Effects of Body Positions on Chest Wall Volume in Abdominal Obesity and Comparison between Abdominal Obesity and Control Groups

The finding in this study showed that both SIT and SWB positions had superior increased total and pulmonary ribcage chest wall volume compared to the two lying positions in the abdominal obesity group. It might be that sitting position related to there being less gravitational compression around the thorax resulting in higher chest wall compliance and lower resistance to diaphragmatic contraction compared to lying down position [18–21, 23]. Furthermore, increasing the pulmonary rib cage volume in sitting positions can be explained by the viscoelastic properties of the ribcage and abdomen region [17]. In sitting positions, the weight of the abdominal content inflates the abdominal wall, and therefore, the compliance of the rib cage is higher than in a supine position [17]. Increase in abdominal region of the chest wall combined with abdominal fat accumulation in the supine position for the abdominal obesity group may be caused by extreme weight of the abdominal content lengthening the diaphragm's fibers, resulting in decreasing the ability of the diaphragmatic muscle contraction.

While the two sitting positions evidenced no significant differences in total chest wall volume, SIT position resulted in higher pulmonary rib cage volume and SWB position resulted in higher abdominal rib cage volume. These differences might be related to the function of the diaphragm. Generally, the diaphragm muscle has two functions, including a respiratory function and a postural stability and core stabilization function [39]. In SIT position, the diaphragm muscle performs both the control core stabilization and respiratory functions. On the other hand, in SWB position, the trunk muscles may have been relaxed due to the presence of the back support, and the diaphragm may then only need to provide a respiratory function. Thus, the sitting with back support position could potentially be used to increase the respiratory function of diaphragm muscle and therefore facilitate diaphragm movement and improve the lung volume when appropriate.

In comparison, our finding that the abdominal obesity group had lower total chest wall volume and its compartments across the body positions than the healthy control group is consistent with that of Barcelar and colleagues who compared these groups with respect to chest wall volume but only in a sitting position [24]. It is possible that excessive fat around the chest wall directly diminishes the chest wall and the possibility of lung expansion [18–21, 23]. However, we found a reduction in both rib cage and abdomen volume in the abdominal obesity group compared with the nonobesity group, while Barcelar and colleagues [24] found only a reduction in rib cage volume and a compensation increase in abdomen volume in their obesity participants compared with a nonobesity group. These differences might be due to differences in the two samples with respect to sex (Barcelar and colleagues' sample included only women), or by differences in the type of obesity (Barcelar and colleagues' sample had both peripheral and abdominal obesity, whereas only men with abdominal obesity were enrolled in the current study). Individuals with abdominal obesity are more likely to have respiratory problems and respiratory-related diseases than those with peripheral obesity [40, 41]. Moreover, previous studies have found that sex differences in chest wall motion were related to the smaller dimensions of the chest wall and motion in women, and a tendency towards a costal breathing pattern, relative to men [22]. In addition, primary prevention and control of abdominal obesity should further be a special concern.

### 4.2. Effects of Body Positions on Lung Function in Abdominal Obesity and Comparison between Abdominal Obesity and Control Groups

The results showed that both sitting positions had the highest lung function, followed by FW and SUP. In the abdominal obesity group, the reductions in lung function with position changes were greater (i.e., 10% to 17%). These results are consistent with those of other researchers who have studied lung function changes associated with position changes (sitting VS. supine positions) in obesity individuals [18–21]. These findings may be due to both gravitational effects combined with a fat mass loading of the thorax and abdomen cavity in obesity, which may limit chest wall expansion, increase thoracic and intra-abdominal pressure, and restrict diaphragmatic excursion during the FVC manoeuvre [18–21, 23]. Furthermore, gravitational force that induces raising venous return to thoracic cavity in lying positions may result in increasing intra-pulmonary blood volume and intrapulmonary airway narrowing and airway resistance [18, 23, 42].

Interestingly, it was not only a fully supine position but also Fowler's position that may reduce lung function, compared with the sitting positions. Although we found that most of the lung function outcomes in Fowler's position showed no significant differences when compared with the sitting positions in the obesity group, FVC in Fowler's position was lower than that in sitting with back support position and FEV1 in Fowler's position was lower than that in both sitting positions (SIT and SWB) in the obesity group. It is possible that a semisitting position may only minimally restrict antero-posterior chest wall expansion and reduce the chest wall compliance. Furthermore, Fowler's position could reduce upper airway diameter which probably increases the upper airway resistance [43, 44]. Thus, while Fowler's position may improve lung function in some domains, relative to supine position, it may also contribute to decreased lung function in other domains, relative to fully sitting positions.

In comparison, the findings that the obesity group had significantly lower lung function across all four body positions than the nonobesity group are consistent with those of other researchers [18–21, 23]. This consistent finding may be explained by the excessive fat around the thoraco-abdominal area, which restricts chest wall and lung expansion, elongates the diaphragmatic and abdominal muscle length, and diminishes the ability of muscle contraction during FVC manoeuvre position [42].

### 4.3. Effects of Body Positions on Respiratory Muscle Strength in Abdominal Obesity and Comparison between Abdominal Obesity and Control Groups

Based on our results, both sitting positions for the abdominal obesity group have higher respiratory muscle strength than both lying positions. This result is caused by the mechanical advantage of the upright position that leads to higher respiratory muscle force and contraction in the sitting position compared with lying positions [18, 19, 21]. Furthermore, our findings indicate that a fully supine position results in the largest reductions in muscle strength in the abdominal obesity group. These results are consistent with the findings from previous researchers [18, 19, 21]. The gravitation effects induced by lying down appear to lead to mechanical disadvantages that reduce the respiratory performance [18, 19, 21].

In comparison to the control group, the abdominal obesity group has lower respiratory muscle strength among body position than the control group but is not significantly different. The finding may be due to excessive fat around ribcage and abdominal area, which elongates the diaphragmatic and abdominal muscles and subsequently affects respiratory muscle contraction [42]. Moreover, previous studies have reported that obesity individuals have lower oxidative capacity, fewer mitochondria, and increased intracellular lipid concentration in skeletal muscles than nonobesity individuals [45]. As a result, individuals with abdominal obesity have lower respiratory muscle strength than nonobesity individuals.

## 5. Limitations

This study had some limitations that should be considered when interpreting the results. First, the study participants were men with abdominal obesity. Research is needed to examine the effects of different types of obesity in samples of women and men to determine which findings from the current study replicate in other groups and which are moderated by sex and obesity type. Second, the study focused on sitting without and with back support, Fowler's, and supine positions. It did not include other positions such as side-lying and prone positions, which are sometimes used to treat acute breathing problems. Future research should include the evaluation of the effects of these positions as well. Third, it is possible, even likely, that other parameters such as chest wall kinematics are influenced by both obesity and body positions. Research is needed to examine these other outcomes as well.

## 6. Conclusions

Consistent with the study hypotheses, the findings suggest that sitting with and without back support positions are the positions that result in the greatest improvements in lung function, total and rib cage chest wall volumes, and inspiratory muscle strength in the abdominal obesity group. Furthermore, the supine position was associated with the greatest reductions in total and rib cage compartmental chest wall volume, lung function, and inspiratory muscle strength. This position should be used with caution when considering implementation into clinical setting. Furthermore, abdominal obesity in men reduces both chest wall volume and lung function compared with lean men and would therefore likely be a high risk respiratory-related illness. Based on our results in asymptomatic males with abdominal obesity, the findings also indicate that further research to evaluate the effects of different types of obesity and different body positions in both men and women on other lung function parameters such as chest wall kinematics, and in samples with respiratory problems, is warranted.

## Figures and Tables

**Table 1:** Baseline characteristics of subjects (*n* = 40).

Characteristics	Mean ± SD		*p* value
	Healthy control (*n* = 20)	Abdominal obesity (*n* = 20)	
Age (years)	27.2 ± 3.90	27.40 ± 4.35	0.879
Weight (kg)	64.23 ± 5.19	85.36 ± 9.19	<0.0001^*∗*^
Height (cm)	171.95 ± 5.32	171.65 ± 4.08	0.842
BMI (kg/m^2^)	21.62 ± 0.95	28.92 ± 2.80	<0.0001^*∗*^
Physical activity (score)	5.16 ± 0.39	5.08 ± 0.67	0.655
Waist circumference (cm.)	76.37 ± 5.57	93.55 ± 20.50	<0.001^*∗*^
Hip circumference (cm.)	91.23 ± 3.81	103.15 ± 6.44	<0.0001^*∗*^
Waist hip ratio (WHR)	0.83 ± 0.03	0.96 ± 0.05	0.0001^*∗*^
Total body fat (%)	15.49 ± 3.96	26.69 ± 3.88	<0.0001^*∗*^
Visceral fat (%)	5.18 ± 1.25	13.29 ± 3.28	<0.0001^*∗*^
Subcutaneous fat (%)	11.27 ± 2.28	19.57 ± 3.39	<0.0001^*∗*^
(i) Arm segment	14.3 ± 4.04	23.35 ± 6.43	<0.0001^*∗*^
(ii) Trunk segment	12.72 ± 4.89	20.05 ± 4.20	<0.0001^*∗*^
(iii) Leg segment	16.25 ± 3.32	26.17 ± 5.19	<0.0001^*∗*^
Truncal skinfold (mm)	57.66 ± 20.63	123.01 ± 23.47	<0.0001^*∗*^
SpO_2_^a^	98.90 ± 0.30	98.7 ± 0.47	0.120
Systolic blood pressure^a^ (mmHg)	111.80 ± 5.65	113.00 ± 3.27	0.417
Diastolic blood pressure^a^ (mmHg)	80.70 ± 2.62	80.80 ± 4.42	0.931
Heart rate^a^ (bpm)	79.2 ± 9.26	83.70 ± 7.90	0.107
Respiratory rate^a^ (bpm)	14.45 ± 1.19	16.5 ± 1.60	<0.0001^*∗*^

^*∗*^Significant difference between abdominal obesity and control groups (*p* < 0.0001). ^a^Measured in high sitting position.

**Table 2:** Effects of abdominal obesity and body positions on lung function.

Parameters	Groups	Body positions				Interaction effects (F3, 36) *p* values
		Sitting (SIT)	Sitting with back support (SWB)	Fowler's position (FW)	Supine (SUP)	
*Lung function*						
FVC (L)	Healthy control	4.53 ± 0.12	4.51 ± 0.11	4.25 ± 0.11^#,*δ*^	4.16 ± 0.11^#,*δ*^	0.001
	Abdominal obesity	3.94 ± 0.12^*∗*^	3.98 ± 0.11^*∗*^	3.76 ± 0.11^*∗*,#,*δ*^	3.65 ± 0.11^*∗*,#,*δ*^	0.001

FEV1 (L)	Healthy control	3.75 ± 0.10	3.73 ± 0.11	3.54 ± 0.11^#,*δ*^	3.39 ± 0.12^#,*δ*^	0.001
	Abdominal obesity	3.23 ± 0.10^*∗*^	3.20 ± 0.11^*∗*^	2.91 ± 0.11^*∗*,#,*δ*^	2.69 ± 0.12^*∗*,#,*δ*,†^	0.001

FEV1/FVC (%)	Healthy control	82.78	82.67	83.29	81.49	
	Abdominal obesity	81.98^*∗*^	80.40^*∗*^	77.39^*∗*,#,*δ*^	73.70^*∗*,#,*δ*,†^	0.001

PEFR (L)	Healthy control	6.49 ± 0.28	6.25 ± 0.25	6.12 ± 0.25	5.74 ± 0.23^#,*δ*,†^	0.001
	Abdominal obesity	5.07 ± 0.28^*∗*^	5.21 ± 0.25^*∗*^	5.02 ± 0.25^*∗*^	4.54 ± 0.23^*∗*,#,*δ*,†^	0.001

ERV (L)	Healthy control	1.12 ± 0.02	1.11 ± 0.37	0.95 ± 0.03	0.90 ± 0.03	
	Abdominal obesity	0.94 ± 0.02^*∗*^	0.92 ± 0.04^*∗*^	0.77 ± 0.03^*∗*^	0.65 ± 0.03^*∗*^	0.001

^*∗*^Significant difference between control and abdominal obesity groups (*p* < 0.001); ^#^significant difference compared with sitting position in the same group; ^*δ*^significant difference compared with sitting with back support in the same group (*p* < 0.001), ^†^significant difference compared with Fowler's position in the same group (*p* < 0.001).

## Data Availability

The experimental quantitative data used to support the findings of this study are included within the article.
